# Polydopamine nanoparticles attenuate retina ganglion cell degeneration and restore visual function after optic nerve injury

**DOI:** 10.1186/s12951-021-01199-3

**Published:** 2021-12-20

**Authors:** Xiaotong Lou, Yuanyuan Hu, Hong Zhang, Jia Liu, Yin Zhao

**Affiliations:** 1 Department of Ophthalmology, Tongji Hospital, Tongji Medical College, Huazhong University of Science and Technology, Wuhan, 430030 China; 2 Research Center for Tissue Engineering and Regenerative Medicine, Union Hospital, Tongji Medical College, Huazhong University of Science and Technology, Wuhan, 430022 China

**Keywords:** Polydopamine nanoparticle, Reactive oxygen species scavenging, Retina ganglion cell, Drug delivery, Optic nerve injury

## Abstract

**Background:**

Oxidative stress contributes to retina ganglion cells (RGCs) loss in variety of ocular diseases, including ocular trauma, ocular vein occlusion, and glaucoma. Scavenging the excessed reactive oxygen species (ROS) in retinal neurovascular unit could be beneficial to RGCs survival. In this study, a polydopamine (PDA)-based nanoplatform is developed to protect RGCs.

**Results:**

The PDA nanoparticles efficiently eliminate multi-types of ROS, protect endothelia and neuronal cells from oxidative damage, and inhibit microglia activation in retinas. In an optic nerve crush (ONC) model, single intravitreal injection of PDA nanoparticles could significantly attenuate RGCs loss via eliminating ROS in retinas, reducing the inflammatory response and maintaining barrier function of retinal vascular endothelia. Comparative transcriptome analysis of the retina implied that PDA nanoparticles improve RGCs survival probably by altering the expression of genes involved in inflammation and ROS production. Importantly, as a versatile drug carrier, PDA nanoparticles could deliver brimonidine (a neuroprotection drug) to synergistically attenuate RGCs loss and promote axon regeneration, thus restore visual function.

**Conclusions:**

The PDA nanoparticle-based therapeutic nanoplatform displayed excellent performance in ROS elimination, providing a promising probability for treating retinal degeneration diseases.

**Graphical Abstract:**

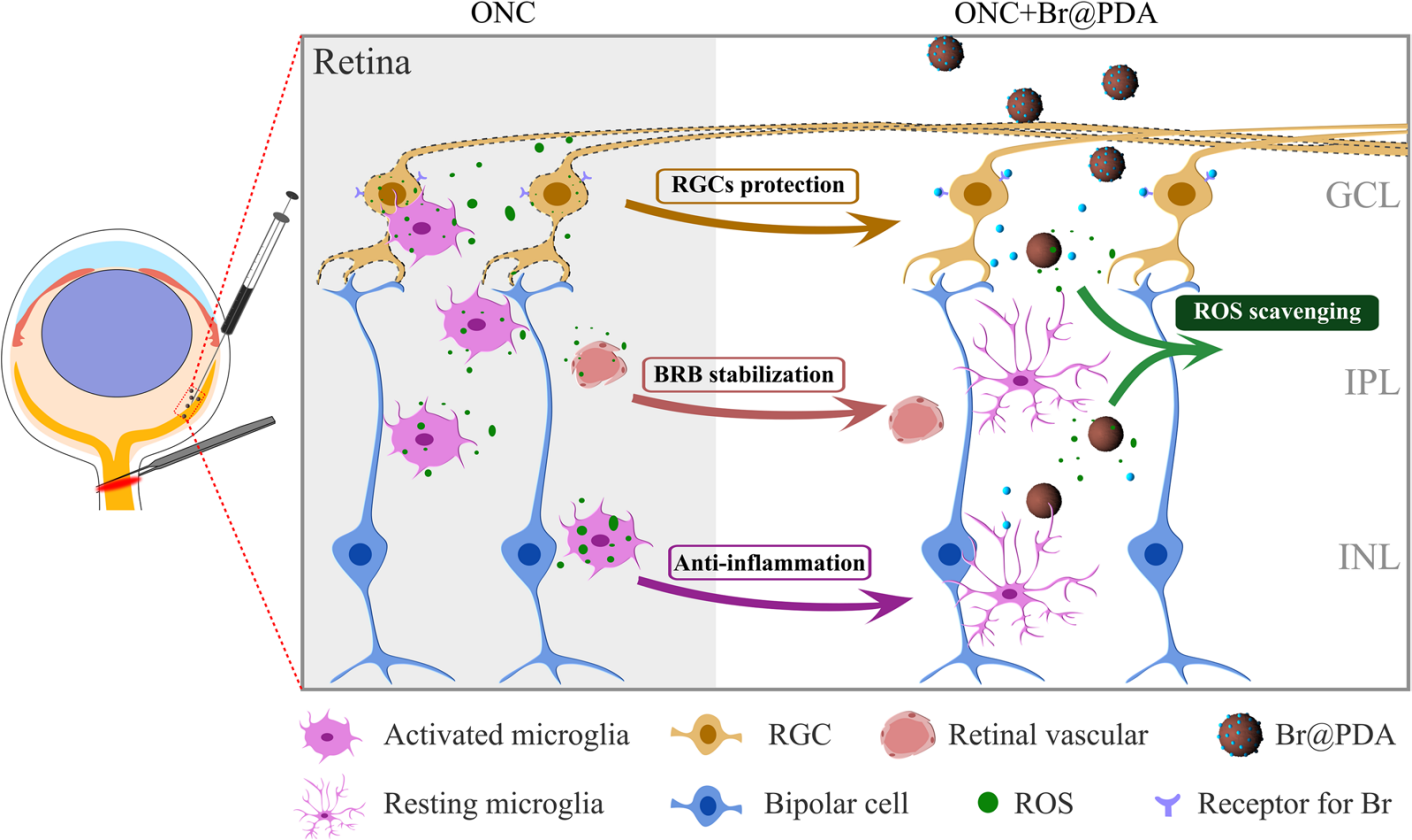

**Supplementary Information:**

The online version contains supplementary material available at 10.1186/s12951-021-01199-3.

## Background

Variety of ocular diseases, including ocular trauma [1], ocular vein occlusion [2], and glaucoma [3] are characterized by retina ganglion cells (RGCs) death, which is the cause of irreversible vision loss [4]. Oxidative stress reflects an imbalance between reactive oxygen species (ROS) production and antioxidant defenses [5, 6]. ROS overproduction is followed by neuronal injury, neuroinflammation and vascular dysfunction in the retinal neurovascular unit, which refers to the functional coupling and interdependency of neurons, glia, and vasculature. Axonal injury is one of the main reasons that triggers RGCs somal loss, as occurs in optic nerve injury or conditions with pathologically increased pressure, e.g., glaucoma [7]. Intracellular superoxide burst has been observed in acute axonal injury [8]. The immoderate ROS induces irreversible oxidative damage on mitochondrial DNA, leading to the apoptosis of RGCs [9, 10]. Thus, reducing ROS accumulation in the retinal neurovascular unit could be beneficial to RGCs survival. Previous studies revealed that deficiency of antioxidant nutrients (e.g. vitamin B1, vitamin E) increased risk of open angle glaucoma, as well as RGCs loss [11, 12]. In addition, antioxidant compounds showed therapeutic potential in neurodegeneration diseases, include coenzyme Q10 [13], *N*-acetyl cysteine [14], acetyl-l-carnitine [15] and alpha lipoic acid [16]. The reduction of oxidative stress is a promising therapeutic approach for RGCs damage [4]. Although gene therapy targets antioxidant enzymes were proposed as a therapeutic option for ocular diseases [17, 18], the poor transfection efficiency in vivo and inflammatory response associated with viral vectors limited its application [19]. Thus, artificial antioxidants, such as Se nanoparticles, metallic-based (e.g., Pt and CeO2), carbon-based (e.g., fullerene and graphene) and polymeric (e.g., hydroxybenzyl alcohol-incorporated polyoxalate copolymer) nanoparticles might serve as an alternative [20, 21].

Polydopamine (PDA), a melanin-like polymer produced by nature neurotransmitter dopamine, has been widely used in biomedical applications because of its excellent biocompatibility, biodegradability, and photothermal transfer ability [22, 23]. Owing to the abundant phenolic groups, PDA possesses ROS scavenging property, and has been applied in alleviating ROS-mediated injury and inflammation [24, 25]. In acute models of peritonitis and lung injury, PDA treatment markedly diminished ROS generation and reduced proinflammatory cytokines [26]. Bao and colleague also reported that PDA could decrease periodontal inflammation [27]. In addition, due to its great drug loading capability through π–π stacking interaction and hydrogen bond, PDA also has been explored as drug carriers [28–30]. Doxorubicin [31], oxaliplatin [32], prostate-specific membrane antigen inhibitor [30] and many other anti-cancer drugs were identified to be encapsulated and delivered by PDA. In glaucoma disease, the negatively-charged microRNA (miR-21-5p) was delivered by cationic PDA nanoparticle (PDA-polyethylenimine) to increase the permeability of outflow pathway, further reduce intraocular pressure (IOP) [33]. Thus, we hypothesized that PDA nanoparticle could serve as a therapeutic nanoplatform to prevent RGCs degeneration via scavenging ROS and delivering therapeutic agents.

Brimonidine, a selective alpha-2 adrenoceptor agonist, is clinically used for reducing IOP [34, 35]. In addition, brimonidine exerts neuroprotective effect by regulating the activity of postsynaptic excitatory *N*-methyl-d-aspartate (NMDA) receptor in RGCs [36–40]. Brimonidine is usually used as topical eye drops (brimonidine tartrate, Alphagan^®^) [41, 42], which however possesses poor drug bioavailability (1–7%) and fast clearance requiring frequently drug administration [43, 44], and even gives rise to incidence of periocular allergic reactions (12.7%) and various side effects (e.g. itching, puffy eye, and shallow breathing) [45]. Hence, directly delivering brimonidine into the vitreous chamber using nanoencapsulation is a promising approach to improve the bioavailability and avoid undesired side effects [46–48].

Herein, we designed a versatile therapeutic platform using PDA nanoparticles for ROS scavenging and brimonidine delivery in preventing RGCs damage. The PDA nanoparticles could sufficiently eliminate multi-types of reactive species, reduce the cellular ROS levels, and protect endothelia and neuronal cells from oxidative damage. In the model of optic nerve crush (ONC), PDA markedly reduced ROS levels in the retinas, and improved RGCs survival probably by altering the expression of genes involved in inflammation and ROS production. Importantly, synergism of the brimonidine-loaded PDA (Br@PDA) provided superior therapeutic efficacy over PDA or brimonidine for attenuating RGC loss and visual function impairment. In summary, our strategy could be considered as a creative inspiration for future neuroprotection drug development.

## Methods

### Synthesis and characterization of PDA nanoparticles

The PDA nanoparticles were synthesized according to previous work [27]. Dopamine hydrochloride (1.0 g) was first dissolved in water (40 mL). Next, this solution was added into a mixture solution (4 mL ammonium hydroxide, 160 mL water, and 80 mL ethanol) and stirred at room temperature for 24 h. the nanoparticles were collected by centrifugation, washed by water, and dried by lyophilization. To prepare brimonidine loaded PDA nanoparticles (Br@PDA), the PDA (10 mg) were dispersed in the methanol containing brimonidine (1 mg/mL, 8 mL). Subsequently, phosphate-buffered saline (PBS, 2 mL) was added, and the dispersion was stirred at room temperature for 24 h. The Br@PDA was collected by centrifugation, washing and lyophilization. The loading content was calculated by determining the remanent brimonidine in the supernatant using a UV–Vis spectrophotometer (Lambda Bio40, PerkinElmer).

The morphology of PDA nanoparticles was observed by transmission electron microscope (FEI TECNAI F20). The hydrodynamic size and zeta potential of PDA nanoparticles were determined using Nano-ZS ZEN3600 (Malvern, UK). Fourier transform infrared spectroscopy (FTIR) was performed in Spectrum One spectrometer (Perkin-Elmer). The content of phenolic groups of PDA nanoparticles was determined using Folin-Ciocalteu assay [49]. The caffeic acid was utilized to established standard curve (760 nm). The phenolic/quinone ratio of PDA nanoparticles was examined by X-ray photoelectron spectroscopy (XPS, Thermo K-Alpha).

### ROS-scavenging effects of PDA nanoparticles

The O_2_^**.**−^ scavenging effect of PDA was examined as previous reported by determining nitro blue tetrazolium (NBT) photoreduction [27]. PDA nanoparticles (0.1 to 1.6 mg/mL) were firstly mixed with riboflavin, methionine, and nitro blue tetrazolium (NBT) in PBS buffer, and then the dispersions were exposed to white light for 5 min. The absorbance of these samples was measured at 560 nm using a microplate reader (Infinite F50).

The ·OH scavenging effect of PDA was examined by determining the oxidation of 3,3ʹ,5,5ʹ-tetramethylbenzidine (TMB). Briefly, PDA dispersions were mixed with hydrogen peroxide, and FeSO_4_ in acetic acid buffer (0.5 M, pH 4.5), and incubated for 10 min. Next, the suspensions were centrifuged, and the supernatants were measured at 652 nm.

The free-radical scavenging effect of PDA was examined as previous work [50]. PDA dispersions were mixed DPPH· in methanol for 20 min. Then, the supernatants were collected by centrifugation and measured at 517 nm.

### Animals

Male C57BL/6 mice (8 weeks) were purchased from Gempharmatech (Nanjing, Jiangsu, China). Animals were fed with standard food and water in a 12-h light/dark cycle. All the animal protocols and procedures were in accordance with National Institutes of Health guide for the care and use of Laboratory animals (NIH Publications No. 8023, revised 1978) and Vision Research and the Use Committee of Huazhong University of Science and Technology.

### Establishment of optic nerve crush model and intravitreal injections

Optic nerve crush model was performed as previously described [51]. Briefly, C57BL/6 mice were anesthetized with intraperitoneal injection of ketamine (100 mg/kg) and xylazine (10 mg/kg). An incision was made in the conjunctiva at the limbus. The left optic nerve was exposed through the muscle cone and crushed at 1 mm from the optic disc for 5 s using forceps. Immediately after crush, intravitreal injection (2 μL) was completed using a Hamilton syringe (Hamilton, Reno, NV). Finally, the incision was sutured and antibiotic drops were administrated on the eye.

### Cell culture

Human umbilical vein endothelial cells (HUVECs), Raw 264.7, N2a, Human retinal pigment epithelium cells (ARPE-19) and 661W were purchased from China Center for Type Culture Collection (CCTCC). HUVECs, Raw 264.7 and N2a were cultured in DMEM (GIBCO, Gaithersburg, MD, USA), while ARPE-19 and 661W in DMEM/F12 supplemented with 10% fetal bovine serum (GIBCO) and 1% penicillin/streptomycin (Invitrogen) at 37 °C with 5% CO_2_.

### In vitro cytotoxicity

In vitro cytotoxicity was detected by CCK-8 assay (Beyotime). Cells were seeded at the density of 1 × 10^4^ cells per well in 96-well plates. Gradient concentrations of PDA nanoparticles were incubated for 24 h, and cells were washed twice with PBS. Fresh media with CCK-8 solution was incubated for 4 h. Finally, absorbance of each well was measured at 450 nm using a microplate reader.

### Nissl staining

Frozen sections from mouse retinas were treated with cresyl violet at room temperature for 5 min, washed in PBS and dehydrated with a graded series of ethanol solutions. Following clearing with xylene, the slices were mounted with neutral balsam and examined with light microscope.

### Propidium iodide (PI) uptake and cell death analysis

Cells grown on glass coverslips (24-well plates) were incubated with PI (5 μM, Beyotime) and hoechst in culture medium for 30 min at 37 °C with 5% CO_2_. After incubation, cells were washed with PBS for 3 times and analyzed using an inverted confocal microscope (Olympus FV3000).

### RT-PCR

Primers for RT-PCR were: 5ʹ-*CTGCAGCACTTGGATCAGGAACCTG*-3ʹ (sense) and 5ʹ-*GGAGTAGCCTGTGTGCACCTGGAA*-3ʹ (antisense) for iNOS; 5ʹ-*TCTCATTCCTGCTTGTGGC-3*ʹ (sense) and 5ʹ-*CACTTGGTGGTTTGCTACG*-3ʹ (antisense) for TNF-a. Primers for murine Gapdh was purchased from Tsingke Biotechnology (Beijing, China). After amplification, the sample was separated on an agarose gel (2%) containing ethidium bromide. The bands densities were measured using Image J.

### Immunofluorescence

Cells grown on glass coverslips were fixed with 4% paraformaldehyde for 15 min at room temperature. After washed twice with PBS, cells were permeabilized with 0.1% Triton X-100 for 10 min, blocked with 5% BSA for 1 h, then incubated with primary antibodies at 4 °C overnight, followed by appropriate secondary antibodies. For tissues, frozen eyes were prepared and sectioned into 10 µm. Frozen sections for immunofluorescence were prepared using the same protocol as for the cells (see above). Images were captured using an inverted confocal microscope.

### Paracellular permeability assay

HUVECs were seeded on the top Transwell chamber with 0.4 μm pore-size membrane (Corning, 3413) and grown for a minimum 2 days until full confluence. Cells were treated with H_2_O_2_ (200 μM) with or without PDA (100 mg/mL) for 6 h at 37 °C, followed by 3 washs with PBS. FITC-dextran of 70 kDa (Sigma, 1 mg/mL) was added to the top chamber. After 1.5 h, the sample was collected from the bottom chamber and read in a fluorescence microplate reader (Synergy2, BioTek, Winooski, VT, USA) at 485/528 nm.

### In situ measurement of ROS

Dihydroethidium (DHE) was used to detect ROS levels in retinas as previously described [52]. Briefly, eyes were embedded (OCT, Tissue-Tek) and frozen in liquid nitrogen immediately after isolation. DHE (10 μmol/L, Beyotime) was applied to 10 μm unfixed cryosections and incubated for 30 min at 37 °C. Images were captured using an inverted confocal microscope.

### Analysis of visual function

Mice were maintained in the testing room for 1 h in dark conditions in their home cage with free access to food and water. Before test, each mouse was allowed to habituate to the testing conditions for 10 min while in the dark.

Optomotor response was analyzed using a testing chamber and software (Softmaze, Shanghai, China). The mice were placed on a platform surrounded by four screens. Vertical sine wave gratings (100% contrast) were projected on the screens. The spatial frequencies tested at a constant speed of 12°/s for 60 s per time and each mice was tested for 10 times. The spatial frequency of the grating was systematically increased until the animal no longer responded. Visual acuity was determined using the threshold of the highest spatial frequency. A more detailed description of the device and methodology is given elsewhere [53].

The light/dark transition test was conducted in an apparatus that consists of a cage (45 * 27 * 27 cm) divided into two chambers of different size (light/dark: 2/1). The light and dark sections were connected by an opening with door (5 * 5 cm). Mice were initially placed into the dark side and the door is opened after the acclimation period and allowed to move freely between the two chambers for 10 min. The time spent in light or dark chambers were recorded and used for analysis.

### Quantification of RGC survival

To estimate the number of surviving RGCs, the retina sections was stained with RBPMS antibody (abcam, ab194213, 1:400). The number of RBPMS-positive cells at central and peripheral retinal were quantified separately. The average of cell counting on each retina was used for analysis. For statistical analysis, the density of RBPMS-positive cells (number of cells per mm) was compared.

### Anterograde labeling and quantification of RGC axons

Following anesthesia, 1 μL cholera toxin subunit B (CTB, 2 μg/μL, BrainVTA) was intravitreally injected with a Hamilton syringe. The mice were sacrificed and 2 days later, mice were deeply anaesthetized and optic nerve segment were dissected, sequentially perfused in 4% PFA, dehydrated in 30% sucrose, then embedded in OCT. Longitudinal sections of 10 μm thickness were made. Regenerating axons were quantified as previously reported [54]. Briefly, the CTB-positive fibers at the indicated distances (0.1 mm, 0.2 mm, 0.3 mm, and 0.4 mm) distal to the crush site were counted. The number of axons in a nerve with a radius of (r) at the point (d) were counted and the thickness of 8 μm (t) were used together to calculate the estimated number of axons. R represents for the width of counted axons. The formula is:

$$ \sum {\text{ad}} = {\pi r}^{2} \times \left( {axon\;number \div R} \right) \div t $$

### RNA-purification and mRNA library construction

Total RNA was extracted from the retinas using Trizol (Invitrogen, Carlsbad, CA, USA) according to manual instruction. RNA was qualified and quantified using a Nano Drop and Agilent 2100 bioanalyzer (Thermo Fisher Scientific, MA, USA). First-strand cDNA was generated using reverse transcription, followed by a second-strand cDNA synthesis. DNA nanoballs (DNBs) were loaded into the patterned nanoarray and single end 50 bases reads were generated on BGIseq500 platform (BGI-Shenzhen, China). cDNA libraries were prepared for sequencing using the Illumina Nextera XT2 DNA Library Prep Kit (Illumina, CAT#FC-131-1024), and 30–40 million paired-end reads (2 × 75 bp) were sequenced for each sample.

### Statistical analyses

All data are presented as the means ± SD from at least three independent experiments. The statistical analyses were performed using the software GraphPad Prism software (version 1.5.2, GraphPad Software Inc.). Comparisons among multiple groups were assessed using one-way analysis of variance (ANOVA) test, as indicated in the figure legends. Comparisons among two groups were assessed using Student’s t test. A value of *P* < 0.05 was considered statistically significant.

## Results

### PDA nanoparticles effectively scavenged reactive oxygen species (ROS)

**Fig. 1:**
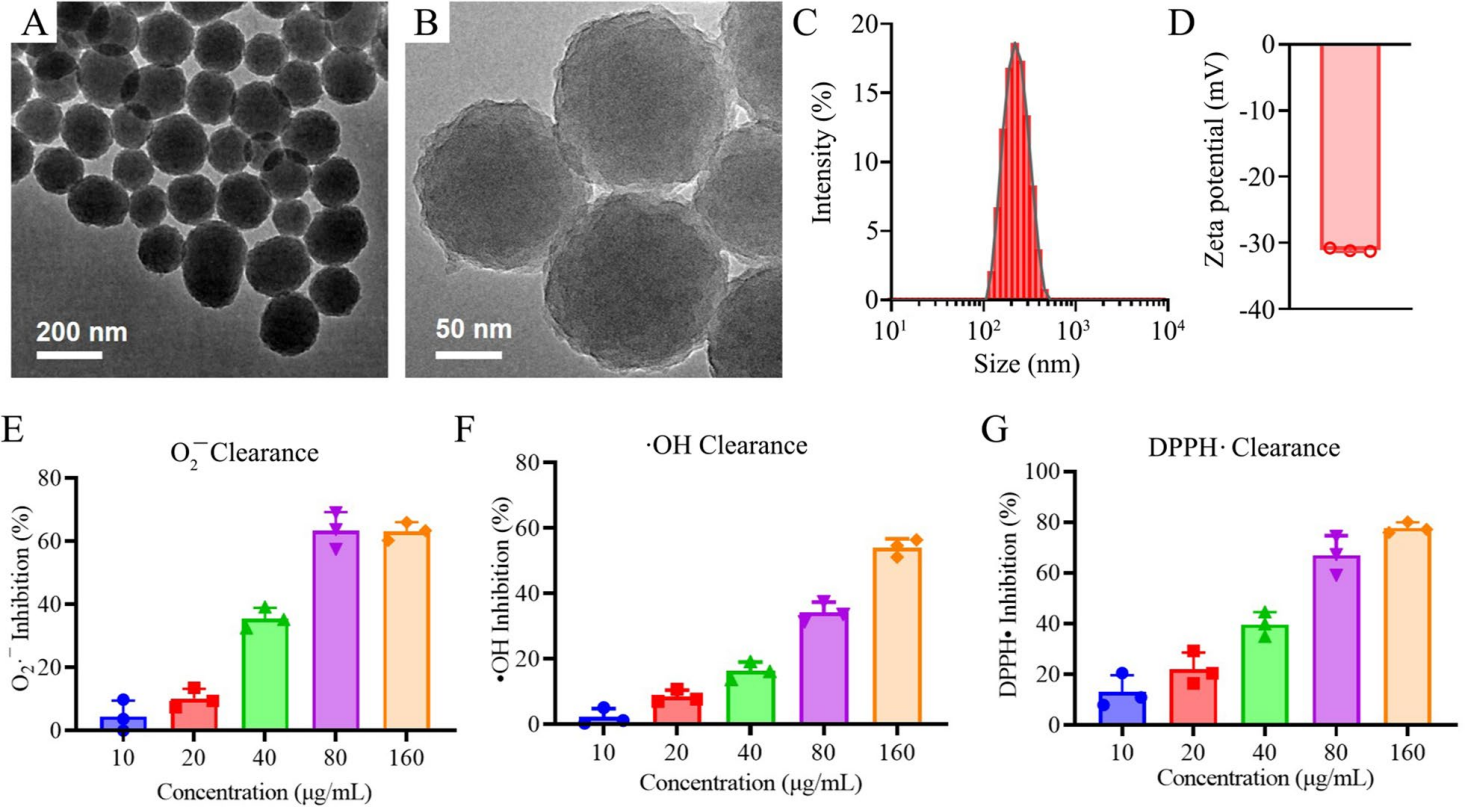
Characterization of PDA nanoparticles. **A**, **B** TEM images of PDA nanoparticles. **C** Hydrodynamic size distribution and **D** zeta-potential of PDA nanoparticles. **E**–**G** ROS scavenging activities of PDA nanoparticles, including **E** superoxide anion (O_2_^•−^), **F** hydroxyl radicals (•OH), **G** DPPH radical

The PDA nanoparticles were synthesized by autooxidation in alkaline solution. Transmission electron microscopy (TEM) revealed that the synthesized PDA possessed spherical morphology (Fig. 1A, B). The PDA nanoparticles were easily dispersed in aqueous solution with a hydrodynamic size of 215 ± 2.6 nm (polydispersity of 0.03) (Fig. 1C), and a negative zeta-potential (− 31 ± 0.27 mV) (Fig. 1D), suggesting that uniform PDA nanospheres were synthesized via self-polymerization of dopamine. The O 1 s XPS spectrum of PDA nanoparticles revealed two characteristic peaks assigned to phenolic C–OH (69%) and quinone C=O (31%) (Additional file 1: Fig. S1). The quantitative phenolic groups in the synthesized PDA nanospheres were further determined to be 1.58 mmol/g, which is associated with the antioxidant properties of PDA [55].

To evaluate the ROS scavenging effects of PDA nanoparticles, the clearance efficacy of superoxide anion (O_2_^**.**−^), hydroxyl radicals (·OH), and DPPH radical were studied. As shown in Fig. 1E and Additional file 1: Fig. S2A, the PDA nanoparticles significantly suppressed the NBT reduction under light irradiation in a dose-dependent manner, suggesting the O_2_^**.**−^ elimination property of PDA. Approximately 60% of O_2_^**.**−^ was removed by PDA at the concentration of 80 μg/mL. Similarly, PDA effectively removed the ·OH by inhibiting TMB oxidation (Fig. 1F and Additional file 1: Fig. S2B), and scavenged DPPH radical dose-dependently (Fig. 1G and Additional file 1: Fig. S2C). These results indicate that PDA nanoparticles could effectively eliminate variety of ROS, and are promising to serve as antioxidant agents for preventing GRCs damage.

### PDA nanoparticles attenuated oxidative damage in endothelia and neuronal cell line

**Fig. 2:**
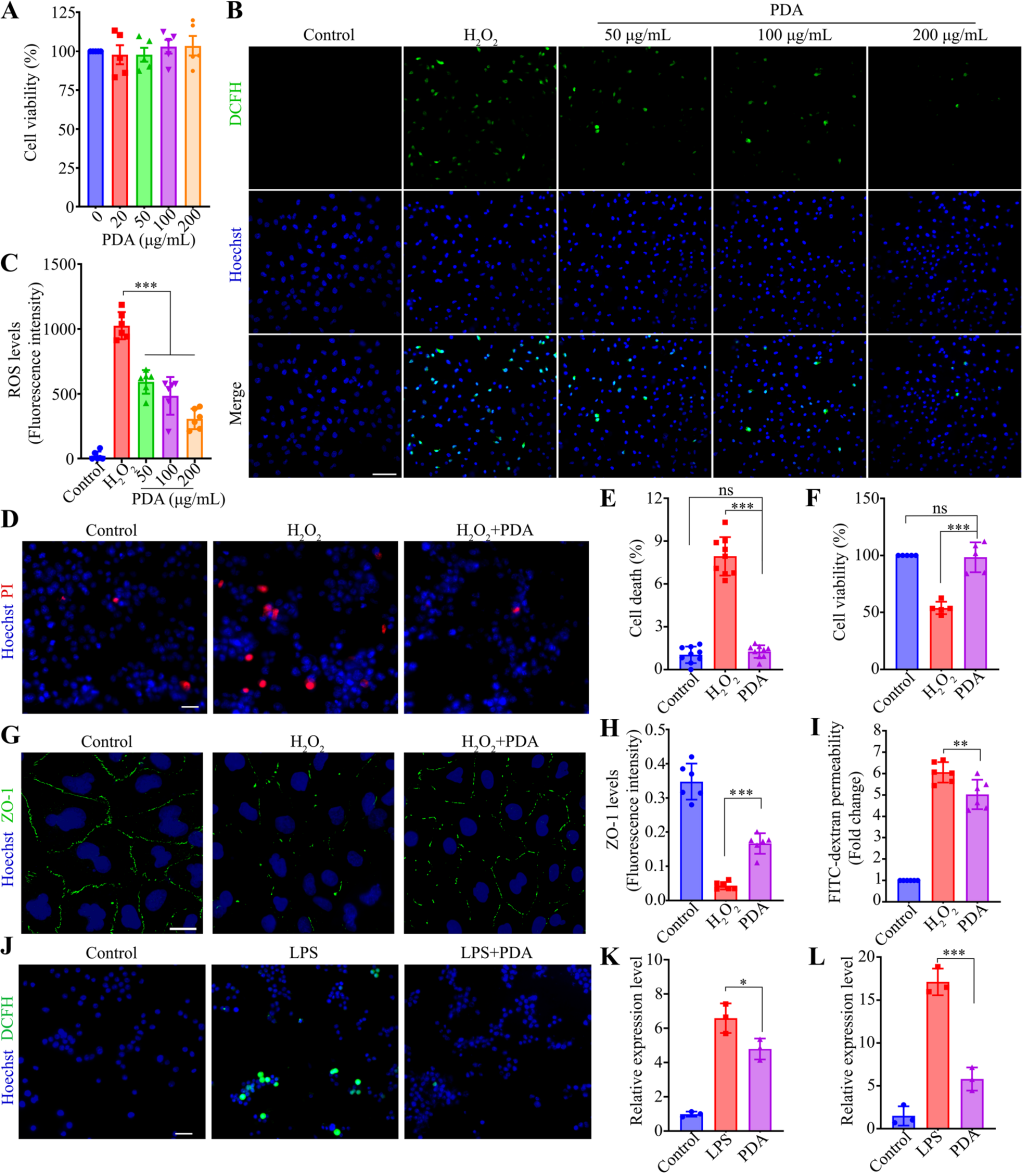
The effects of PDA on ROS levels, cell survival and macrophage polarization in vitro. **A** The cell viability of HUVECs treated with different concentrations of PDA (0, 20, 50, 100 and 200 μg/mL) was determined by CCK-8 assay. n = 5. **B**, **C** HUVECs were treated with H_2_O_2_ (200 μM) and PDA (50, 100 and 200 μg/mL) for 6 h. ROS levels was assayed by DCFH-DA. Representative images and quantitative analysis (ANOVA) of ROS levels were shown. Scale bar, 100 μm. n = 6. **D**, **E** N2a was treated with H_2_O_2_ (200 μM) and PDA (200 μg/mL) for 6 h. PI staining was performed to evaluate cell death. Representative images and quantitative analysis (ANOVA) of PI signals. Scale bar, 40 μm. n = 9. **F** The cell viability of N2a was determined by CCK-8 assay. n = 5. **G**, **H** HUVECs were treated with H_2_O_2_ and PDA (200 μg/mL) for 6 h. Representative images and quantitative analysis (ANOVA) of ZO-1 levels in HUVECs. Scale bar, 30 μm. n = 6. **I** Quantitative analysis (ANOVA) of FITC-dextran permeability in HUVEC monolayer. n = 6. **J** Raw264.7 was treated with LPS (1 μg/mL) and PDA (200 μg/mL) for 12 h. Representative fluorescent microscopy images showing intracellular ROS detected by DCFH in Raw264.7. Scale bar, 40 μm. **K**, **L** PCR was performed to detect the mRNA levels of iNOS and TNF-α in RAW264.7. n = 3. *P < 0.05, **P < 0.01, ***P < 0.001. Data are presented as the mean ± SD

The cytocompatibility of PDA nanoparticles was tested in human umbilical vein endothelial cells (HUVECs) using CCK-8 assay. Exposed to different concentration of PDA (20 to 200 mg/mL) for 24 h, the viability of HUVECs was all higher than 95%, suggesting the negligible cytotoxicity of PDA nanoparticles (Fig. 2A). Moreover, the Human retinal pigment epithelium (ARPE-19) cells and murine photoreceptor cells (661W) were treated with different doses of PDA nanoparticles for 24 or 72 h, and no significant cytotoxicity were observed (Additional file 1: Fig. S3A–H). As indicated by the fluorescence of DCFH, H_2_O_2_ treatment (200 μM) significantly induced oxidative stress in HUVECs, as plenty of fluorescent spots were observed (Fig. 2B). Of note, the intracellular ROS levels of treated HUVECs were markedly decreased by PDA nanoparticles in a dose-dependent manner, confirming the PDA’s ROS clearance activity (Fig. 2B, C). Due to the ROS elimination effect, PDA nanoparticles effectively reduced the H_2_O_2_-induced cell death in neuronal cells (N2a) (Fig. 2D, E), correspondingly raised the cell viability as high as untreated cells (Fig. 2F), suggesting the cyto-protection effect of PDA against ROS.

The homeostasis of the retina is maintained by the blood–retina-barrier (BRB), a complex of different cell types. Junctions between vascular endothelia were essential for the integrity of the BRB. The localization of the tight junction protein zonula occludens-1 (ZO-1) was examined to evaluate junction loss in HUVECs. H_2_O_2_ treatment significantly decreased ZO-1 in HUVECs, while PDA nanoparticles markedly diminished the ZO-1 reduction (Fig. 2G, H), and partially restored the barrier function by reducing the permeability of HUVECs monolayers to macromolecule (FITC-Dextran, 70 kDa) (Fig. 2I). Together, these results indicate that PDA nanoparticles could effectively scavenge ROS, protect cells from ROS-induced damage, and recover the function of different cell types.

### PDA nanoparticles suppressed macrophages polarization

The ROS elimination property of PDA nanoparticles was also assessed in lipopolysaccharide (LPS) stimulated macrophages. Compared with PBS treated cells, LPS (1 μg/mL) treatment markedly elevated the ROS level in RAW264.7 cells (Fig. 2J and Additional file 1: Fig. S4), and dramatically increased the mRNA levels of iNOS and TNF-α (Fig. 2K, L), which indicate the pro-inflammatory polarization tendency of LPS-treated macrophages [56]. In the presence of PDA nanoparticles, the ROS content of LPS-treated RAW264.7 cells was significantly reduced, and the mRNA levels of iNOS and TNF-α were also markedly decreased in LPS-treated cells (Fig. 2J, L), indicating that PDA eliminated the cellular ROS and attenuated M1 polarization.

### PDA nanoparticles attenuated retinal degeneration, and suppressed the activation of microglia after ONC

**Fig. 3:**
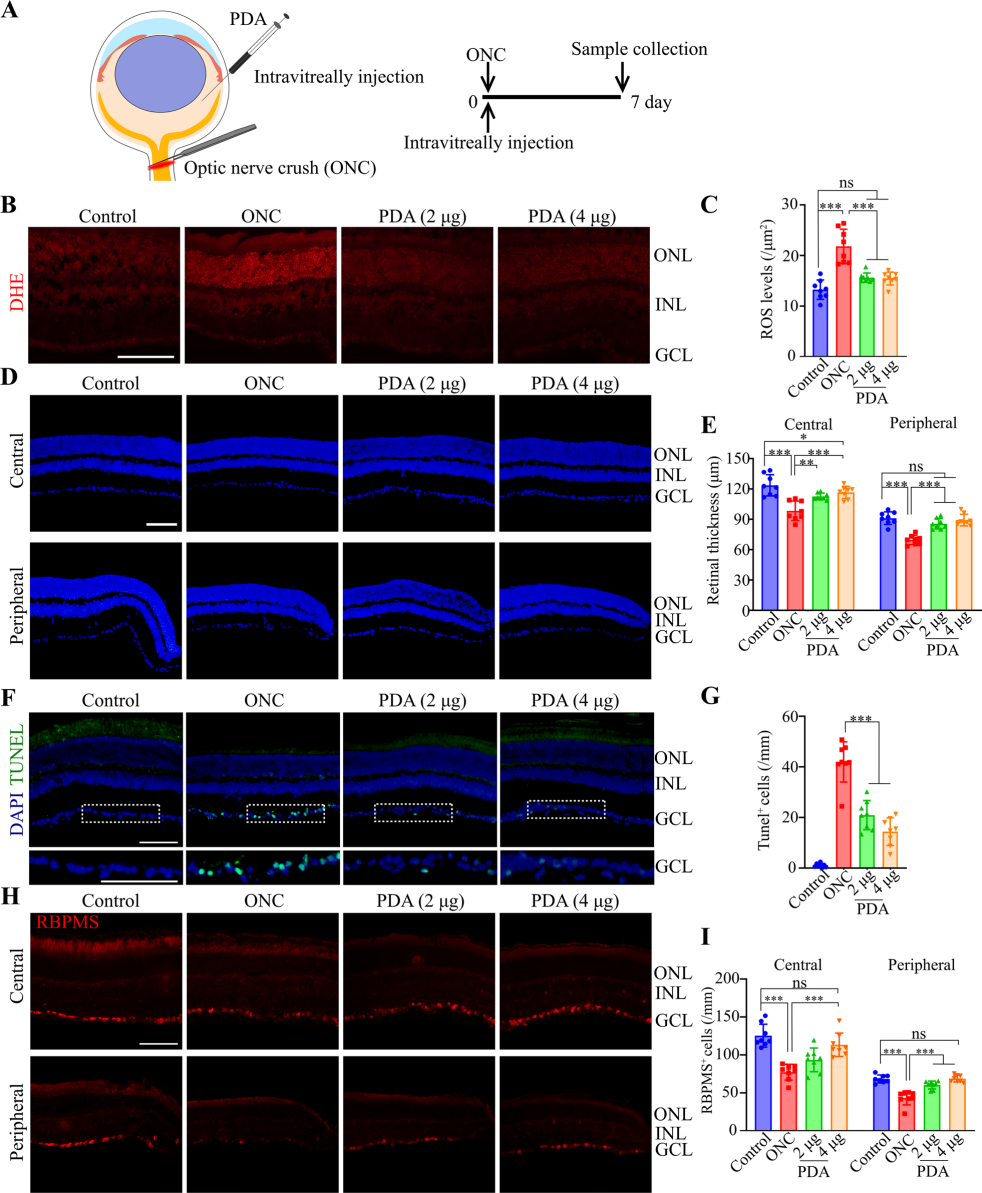
The effects of PDA on retinal thickness and neronal loss in ONC models. **A** Schematic of intravitreally injection of PDA (2 or 4 μg) and the time line for the experiments shown in (**B**–**I**). **B**, **C** Superoxide anion production was detected by DHE. Representative images and quantitative analysis (ANOVA) of ROS levels in retinal sections. Scale bar, 50 μm. n = 8. **D**, **E** Representative images and quantitative analysis (ANOVA) of central and peripheral retinal thickness. Scale bar, 50 μm. n = 8. **F**, **G** Representative image and quantitative analysis (ANOVA) of TUNEL signals. Scale bar, 50 μm. n = 8. **H**, **I** Retinal sections were immunostained for RBPMS (marker of RGCs). Representative images and quantitative analysis (ANOVA) of RGCs density in central and peripheral retinas. Scale bar, 50 μm. n = 8. *P < 0.05, **P < 0.01, ***P < 0.001. Data are presented as the mean ± SD

The in vivo biocompatibility of PDA nanoparticles was first evaluated by intravitreous injection (4 μg) in healthy mice. At day 7 post-injection, the number of apoptotic cells in retinas is not increased by PDA treatment, suggesting the good compatibility in vivo (Additional file 1: Fig. S3I, J). Since superoxide generation was an early event in axonal injuries [8], we then investigated the therapeutic effect of PDA nanoparticles in an optic nerve crush model. PDA nanoparticles were administrated immediately after ONC, and the retinas were collected at day 7 (Fig. 3A). PDA nanoparticles (2 or 4 µg) were able to reduce the retinal ROS to the same level of control (uncrushed) group as indicated by dihydroethidium (DHE) staining (Fig. 3B, C). The thickness from the ganglion cell layer (GCL) to outer nuclear layer (ONL) was evaluated by DAPI staining (Additional file 1: Fig. S5), which revealed the decreased thickness of central and peripheral retinal after ONC. Impressively, PDA nanoparticles markedly protected central and peripheral retinal layers, as the both layers recovered as thick as the control group (Fig. 3D, E). In addition, PDA nanoparticles reduced the neuronal loss in GCL (Additional file 1: Fig. S6). Further, the apoptotic cells and the number of RGCs in GCL were evaluated by TUNEL and RBPMS staining. As shown in Fig. 3F, ONC significantly induced cell apoptosis in the GCL, while PDA nanoparticles markedly decreased the TUNEL positive cells (Fig. 3F, G), suggesting the cyto-protection effect. Moreover, PDA treatments elevated the number of RBPMS-positive cells at both central and peripheral retinal to the same level with control (uncrushed) group (Fig. 3H, I).

Microglia was the tissue macrophage population of the central nervous system. It was identified that reactive microglia are neurotoxic [57, 58], thus we visualized the microglia morphology using ionized calcium binding adaptor molecule 1 (IBA1). Undergoing ONC, the number of IBA1-positive cells with amoeboid-like morphology dramatically increased in retina, while PDA nanoparticles significantly attenuated the microglia infiltration (Additional file 1: Fig. S7), suggesting that PDA suppresses ONC-induced microglia activation in the retina. These results revealed that PDA nanoparticles can effectively eliminate the excessively increased ROS, reduce the retinal neuronal degeneration, and suppress microglia activation after ONC.

### Comparative transcriptome analysis of the retina after ONC treated with or without PDA nanoparticles

**Fig. 4:**
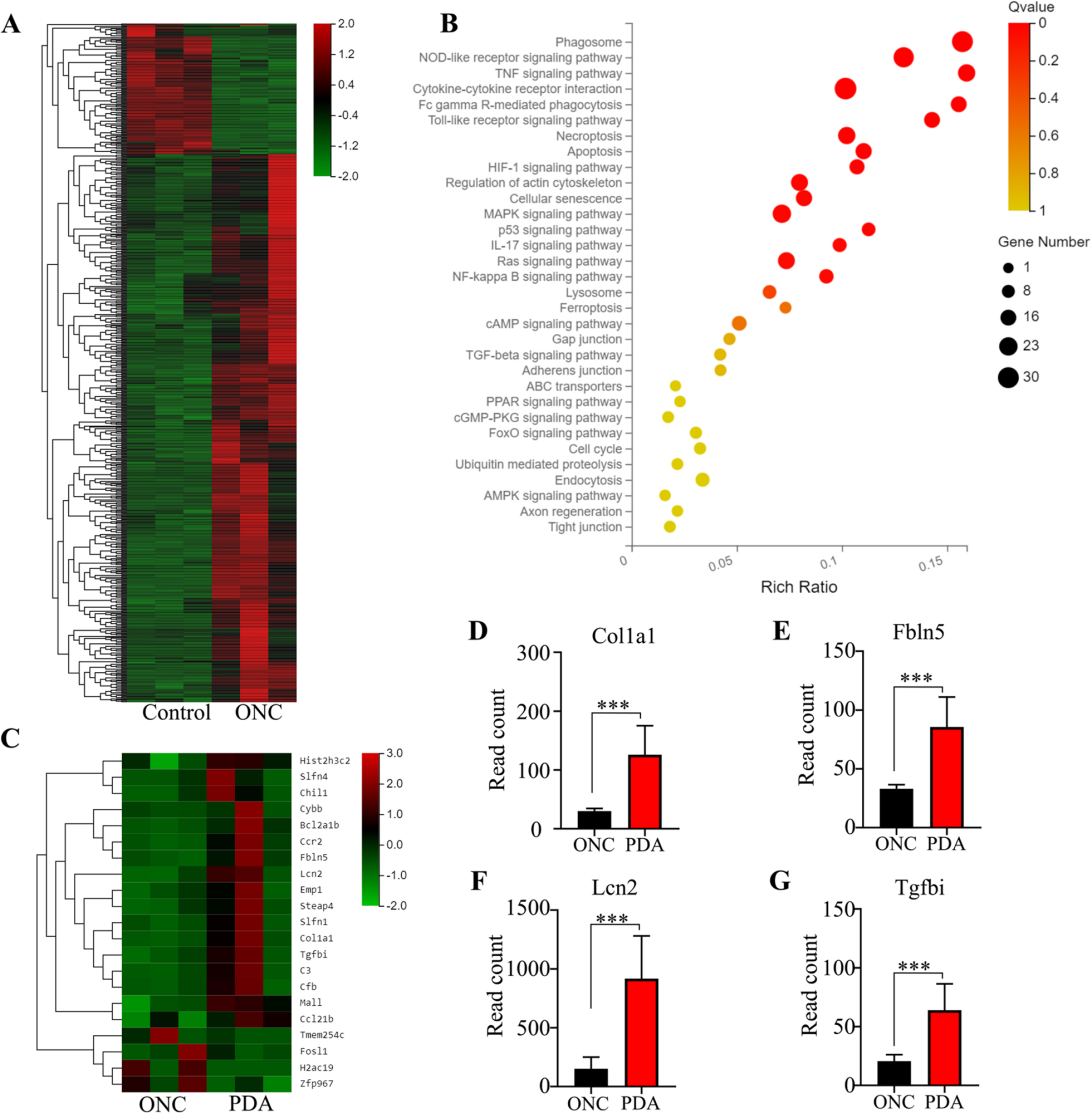
Global transcriptome alterations identified by RNA-seq in retinas of PDA-administrated mice. **A** Heatmap representing the z-score of expression levels of 717 DEG in retinas between control and ONC groups. **B** The KEGG enrichment for the 717 DEG was evaluated by Rich ratio, Q value and the number of genes enriched in the related pathway. **C** Heatmap representing the z-score of expression levels of 21 DEG (total 105) between ONC and PDA groups. **D**–**H** Histogram illustrating raw gene expression values for oxidative associated genes in DEG between ONC and PDA groups. ***P < 0.001. n = 3. Data are presented as the mean ± SD

To investigate the alternations in the retina after ONC and explore the mechanism of neuroprotective effect of PDA nanoparticles, we performed RNA-seq analysis of retinas from healthy mice and ONC mice with or without PDA treatments. We analyzed differentially expression genes (DEGs), and identified 717 genes related to optic nerve injury. The heat map of all differentially expressed genes between the control and ONC groups were shown in Fig. 4A. KEGG enrichment analysis was demonstrated with scatter plots. The enriched pathways of ONC-related DEGs included phagosome (n = 28), NOD-like receptor (n = 26), TNF (n = 18), cytokine–cytokine receptor interaction (n = 30), Fc gamma R-mediated phagocytosis (n = 14), Toll-like receptor (n = 14), necroptosis (n = 18), and apoptosis (n = 15) pathways, which were mainly associated with inflammatory process and cell survival (Fig. 4B). Moreover, the DEGs between PDA and ONC groups were showed in heatmap (Fig. 4C). Col1a1, Fbln5, Lcn2, and Tgfbi were identified as potential oxidative stress-associated genes involved in the process of RGCs protection by PDA nanoparticles (Fig. 4D–F). Compared to the ONC group, PDA nanoparticles significantly elevated the expression of these genes which tend to attenuate oxidative damage [59–62].

### Long-term therapeutic efficacy of brimonidine-loaded PDA nanoparticles

**Fig. 5:**
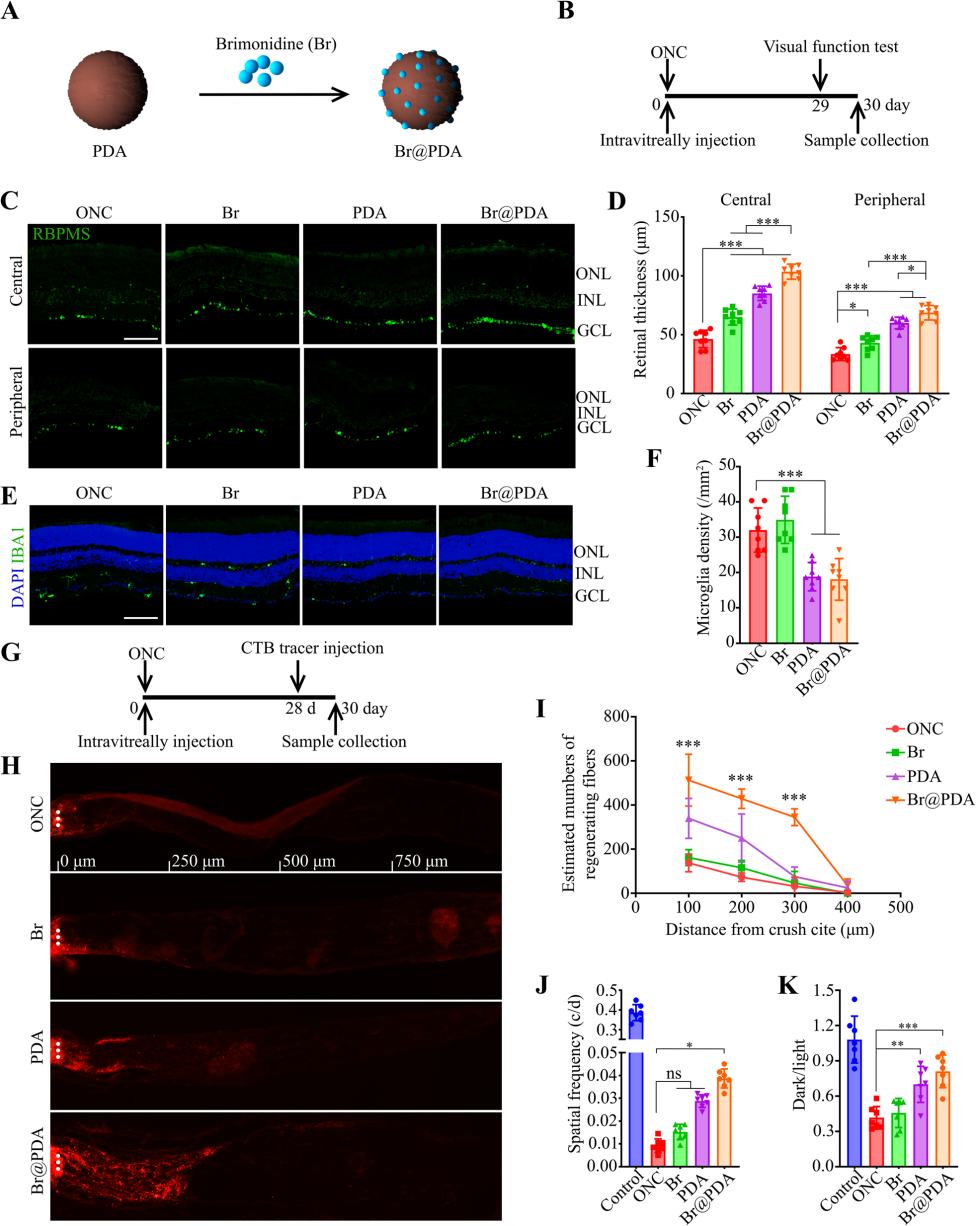
The effects of Br@PDA on RGC survival and axon regeneration 30 days after ONC. **A** Schematic of particles design. **B** The time line for the experiments shown in (**C**–**F**, **J** and **K**). **C**, **D** Retinal sections were immunostained for RBPMS. Representative images and quantitative analysis (ANOVA) of RGC density in central and peripheral retinas. Scale bar, 50 μm. n = 8. **E**, **F** Retinal sections were immunostained for IBA1. Representative images and quantitative analysis (ANOVA) of microglia density in retinas. Scale bar, s50 μm. n = 8. **G** The time line for the experiments shown in (**H**, **I**). **H**, **I** Longitudinal sections of the optic nerve showing CTB-labeled axons at 100, 200, 300, 400, 500 μm distal to the injury site. Representative images and quantitative analysis (ANOVA) of CTB signals. ***P < 0.0001 versus ONC group. n = 6. **J** Quantification (ANOVA) of the optomotor response of mice treated with Br@PDA. n = 7. **K** Quantification (ANOVA) of the ratio of time mice spent in the dark chamber and light chamber. n = 7. *P < 0.05, **P < 0.01, ***P < 0.001. Data are presented as the mean ± SD

The PDA nanoparticles were further served as drug carriers to encapsulate brimonidine (loading content, 20.0%), a selective alpha-2 adrenoceptor agonist that exerts neuroprotective effect by regulating the activity of NMDA receptor in RGCs [37] (Fig. 5A). Brimonidine loading slightly increased the hydrodynamic size (223.9 ± 4.7 nm) and zeta-potential (− 28.5 ± 0.58 mV) of nanoparticles, which remained the spherical morphology (Additional file 1: Fig. S8). Moreover, the FTIR spectra of nanoparticles were hardly changed, likely because the absorption peaks of brimonidine were overlapped with that of PDA (Additional file 1: Fig. S9). Next, the long-term therapeutic effects of brimonidine-loaded PDA (Br@PDA) against optic nerve injury were investigated (Fig. 5B). Compared with the ONC group, both brimonidine and PDA treatments significantly increased the density of RGCs at central and peripheral retinal (Fig. 5C, D; Additional file 1: Fig. S10). Of note, the Br@PDA elevated the RGCs density more effectively than brimonidine and PDA groups, suggesting the combinational therapy effect of Br@PDA. Moreover, Br@PDA significantly decreased the number of microglia as well as PDA alone, likely due to the antioxidant property of PDA, while brimonidine did not affect the microglia infiltration (Fig. 5E, F).

Next, the axon regeneration was detected by intravitreally injection of cholera toxin b-subunit (CTB), a highly sensitive retrograde neuroanatomical tracer (Fig. 5G). As shown in Fig. 5H, PDA nanoparticles significantly increased the density of regenerated axons, while brimonidine hardly enhanced axons regeneration. Importantly, Br@PDA dramatically promoted the axons density, and the regenerated axons were tenfold more than the other groups (ONC, Br and PDA groups) at 0.3 mm distal to the lesion site (Fig. 5H, I), suggesting the synergistic therapy effect of Br@PDA. We further performed the optomotor response test and light/dark transition assay to evaluate the visual acuity and dark preference of mice. Br@PDA group displayed significantly better optomotor response than the ONC group, nevertheless brimonidine or PDA alone ineffectively elevated the threshold spatial frequency of mice (Fig. 5J). The ratio of time spent in dark and light chambers was reduced after ONC, indicating injured ability of photoperceptive. Administration of PDA and Br@PDA elevated the dark/light ratio and no significant difference was found between the two groups (Fig. 5K). These results demonstrated that Br@PDA provided long-term protection from RGC loss and visual function impairment in ONC model.

## Discussion

Oxidative stress damage raised by reactive oxygen species (ROS) is a common and severe pathological process in various diseases. In ocular diseases, such as ocular trauma, ocular vein occlusion and glaucoma, excessed ROS induces irreversible damage of RGCs. Unfortunately, no matter pharmacological or surgical treatment failed to delay the progression of visual field loss in some individuals [63, 64]. This suggests that preclinical drug discovery for neuroprotection in ocular diseases is urgent [65–67]. In this study, we proposed a novel approach for RGCs and optic nerve protection using polydopamine (PDA) nanoparticles-based nanoplatform. For the first time, to our knowledge, we achieve significant visual recovery in optic nerve crush (ONC) model by nanomaterial therapy through ROS elimination. Unlike other preclinical drugs where target one protein, molecular pathway or/and cellular type, the PDA nanoparticles allow single injection to promote RGCs survival, suppress retinal inflammation, and stabilize the barrier function of vascular endothelia cells. Furthermore, as a drug carrier, PDA nanoparticles allow loading neuroprotection drug such as brimonidine to restore visual function synergistically.

PDA is a major pigment of naturally occurring melanin and possesses outstanding biocompatibility [68, 69]. Numerous studies had identified the therapeutic potential of PDA nanoparticles in different diseases, including cancer [70], diabetes [71], inflammation [72] and many other diseases [73, 74]. In this study, we firstly employed PDA nanoparticles for neuroprotection in a model of axonal injury. We found that single intravitreal injection of PDA nanoparticles could scavenge ROS in retina, therefore rescuing the injured RGCs after ONC. In the RGCs, p53, along with Nrf2/HO-1 antioxidant pathway, NF-κB and JAK–STAT3 pathway were identified involved in ROS-induced RGCs apoptosis [75–78]. We assumed that PDA nanoparticles might protect RGCs from ROS induced apoptosis through these pathways.

In addition to scavenge ROS in RGCs, PDA nanoparticles could also reduce the activation of microglia and maintain the barrier function of vascular endothelial cells after ONC. Previous studies reported that the accumulation of ROS can trigger microglia activation [58], then activated microglia will release more ROS to aggravate neurodegeneration [57]. In microglia, ROS induces p65 nuclear translocation therefore regulates microglia polarization by increasing TNF-α, IL-1β and IL-6 secretion [79]. In endothelial cells, the generation of ROS disrupts adherens junctions (AJ) and tight junctions (TJ). For example, Chattopadhyay et al. found that ROS stimulation induced AJ protein tyrosine phosphorylation and AJ disruption [80]. Qin and colleague reported that ROS stimulation downregulates TJ protein expression, such as occludin and zonula occludens [81].

The data of retinal RNA-seq demonstrated that PDA nanoparticles could increase the expression of Col1a1, Fbln5, Lcn2 and Tgfbi, which are found to reduce ROS levels and oxidative damage [59–62]. Fbln5 competed with fibronectin (FN) for binding to α5β1 integrin, resulting in reduced FN-integrin mediated ROS production [59]. LCN2, functioned as an iron transporter as well as an antioxidant. Absence of LCN2 elicited intracellular iron accumulation, thus leading to iron-related oxidative stress [61, 62]. The alternation of LCN2 in retinas implied that iron metabolism might be involved in the neuroprotection and anti-inflammation process of PDA nanoparticles. However, more experiments are needed to identify the detailed mechanism.

As an emerging polymer material, PDA nanoparticles are also used as promising drug carriers in cancer therapy. Comparing with traditional chemotherapy, PDA nanoparticles can target tumor sites through the enhanced permeability and retention effect, therefore reducing the side effects [29]. Furthermore, the surface of PDA nanoparticles can be modified by –SH or –NH_2_ terminated ligands to enhance targeting capability [82]. Previous experimental-glaucoma models showed that brimonidine treatment significantly reduced RGCs apoptosis by 97.7% and 92.8% at 3 and 8 weeks, respectively [83]. In retinal neuronal cells exposed to UV, brimonidine could increase cell viability at 10 and 100 μM. Hence, brimonidine was loaded in PDA nanoparticles (Br@PDA) as a neuroprotection nano-therapeutic to enhance RGCs protection. Notably, the Br@PDA could synergistically rescue RGCs apoptosis, promote optic nerve transport, and improve visual impairment in ONC model.

## Conclusions

In conclusion, we developed a novel approach to decrease RGCs apoptosis induced by ONC. Single intravitreal injection of PDA nanoparticles could efficiently remove ROS in retina, therefore improve neurodegeneration. We also proved the enhanced therapeutic efficiency of neuroprotection drug loaded PDA nanoparticles on visual impairment. These results provide a direction for future translation research for ocular neurodegeneration diseases.

## Supplementary Information

**Additional file 1: Fig. S1.** O 1s XPS spectrum of PDA nanoparticles. **Fig. S2.** Scavenging efficiencies of (A) superoxide anion (O_2_^**.**−^), (B) hydroxyl radicals (·OH), and (C) DPPH radical with different concentrations of PDA nanoparticles. **Fig. S3. **Biocompatibility of PDA nanoparticles in vitro and in vivo. (A–D) The cell viability of 661W (A, B) and ARPE-19 (C, D) treated with different concentrations of PDA (0, 20, 50, 100 and 200 μg/mL) for 24 h and 72 h, which was determined by CCK-8 assay (n = 5). (E–H) Live/Dead cell staining of 661W (E, F) and ARPE-19 (G, H) cells treated with PDA (200 μg/mL) for 24 h or 72 h. Scale bar, 100 μm. n = 5. (I–J) PDA nanoparticles (4 μg) were intravitreously injected in the healthy mice, and the number of apoptotic cells in retinas is evaluated by TUNEL at day 7 post-injection. Scale bar, 50 μm. n = 8. Data are presented as the mean ± SD. **Fig. S4. **Quantitative analysis (ANOVA) of ROS levels in Raw 264.7 treated with LPS (1 μg/mL) and PDA (200 μg/mL) for 12 h. ***P < 0.001. n = 5. Data are presented as the mean ± SD. **Fig. S5. **DAPI staining of the retinal cross section, dashed lines indicate the region for analysis of central and peripheral retinal thickness. Scale bar, 200 μm. **Fig. S6. **Representative images of nissl stained retinal sections in mice treated with PDA (2 μg or 4 μg). Scale bar, 50 μm. **Fig. S7. **Representative imageS and quantitative analysis (ANOVA) of microglia (IBA1-positive) densities in retinal sections. Scale bar, 50 μm. ***P < 0.001. n = 8. Data are presented as the mean ± SD. **Fig. S8. **TEM images of Br@PDA. **Fig. S9. **FTIR spectra of Br, PDA and Br@PDA. **Fig. S10. **Representative image of nissl stained retinal sections in mice treated with PDA, Br and Br@PDA. Scale bar, 50 μm.

## Data Availability

All data generated or analysed during this study are included in this published article and its additional information files.

## Abbreviations

RGCs: Retinal ganglion cells
ROS: Reactive oxygen species
PDA: Polydopamine
ONC: Optic nerve crush
HUVECs: Human umbilical vein endothelial cells
BRB: Brain retinal barrier
LPS: Lipopolysaccharide
DHE: Dihydroethidium
GCL: Ganglion cell layer
ONL: Outer nuclear layer
IBA1: Ionized calcium binding adaptor molecule 1
DEGs: Differentially expression genes
CTB: Cholera toxin subunit B
