# Supplementary material for: Polydopamine nanoparticles attenuate retina ganglion cell degeneration and restore visual function after optic nerve injury
Source: J Nanobiotechnology. 2021 Dec 20;19:436. doi: 10.1186/s12951-021-01199-3 (PMC8686547; doi:10.1186/s12951-021-01199-3)
Supplement: Supplementary file 1 — Additional file 1: Fig. S1. O 1s XPS spectrum of PDA nanoparticles. Fig. S2. Scavenging efficiencies of (A) superoxide anion (O2.−), (B) hydroxyl radicals (·OH), and (C) DPPH radical with different concentrations of PDA nanoparticles. Fig. S3. Biocompatibility of PDA nanoparticles in vitro and in vivo. (A–D) The cell viability of 661W (A, B) and ARPE-19 (C, D) treated with different concentrations of PDA (0, 20, 50, 100 and 200 μg/mL) for 24 h and 72 h, which was determined by CCK-8 assay (n = 5). (E–H) Live/Dead cell staining of 661W (E, F) and ARPE-19 (G, H) cells treated with PDA (200 μg/mL) for 24 h or 72 h. Scale bar, 100 μm. n = 5. (I–J) PDA nanoparticles (4 μg) were intravitreously injected in the healthy mice, and the number of apoptotic cells in retinas is evaluated by TUNEL at day 7 post-injection. Scale bar, 50 μm. n = 8. Data are presented as the mean ± SD. Fig. S4. Quantitative analysis (ANOVA) of ROS levels in Raw 264.7 treated with LPS (1 μg/mL) and PDA (200 μg/mL) for 12 h. ***P < 0.001. n = 5. Data are presented as the mean ± SD. Fig. S5. DAPI staining of the retinal cross section, dashed lines indicate the region for analysis of central and peripheral retinal thickness. Scale bar, 200 μm. Fig. S6. Representative images of nissl stained retinal sections in mice treated with PDA (2 μg or 4 μg). Scale bar, 50 μm. Fig. S7. Representative imageS and quantitative analysis (ANOVA) of microglia (IBA1-positive) densities in retinal sections. Scale bar, 50 μm. ***P < 0.001. n = 8. Data are presented as the mean ± SD. Fig. S8. TEM images of Br@PDA. Fig. S9. FTIR spectra of Br, PDA and Br@PDA. Fig. S10. Representative image of nissl stained retinal sections in mice treated with PDA, Br and Br@PDA. Scale bar, 50 μm. [file 12951_2021_1199_MOESM1_ESM.docx]

**Additional Information**

**Polydopamine nanoparticles attenuate retina ganglion cell degeneration and restore visual function after optic nerve injury**

Xiaotong Lou^1^, Yuanyuan Hu^1^, Hong Zhang^1^, Jia Liu^2^*, Yin Zhao^1^*

1 Department of Ophthalmology, Tongji Hospital, Tongji Medical College, Huazhong University of Science and Technology, Wuhan 430030, China.

2 Research Center for Tissue Engineering and Regenerative Medicine, Union Hospital, Tongji Medical College, Huazhong University of Science and Technology, Wuhan, 430022, China.

* Correspondence should be addressed to: Jia Liu (Email: jialiu1207@hust.edu.cn);

Yin Zhao (Email: zhaoyin85@hust.edu.cn)


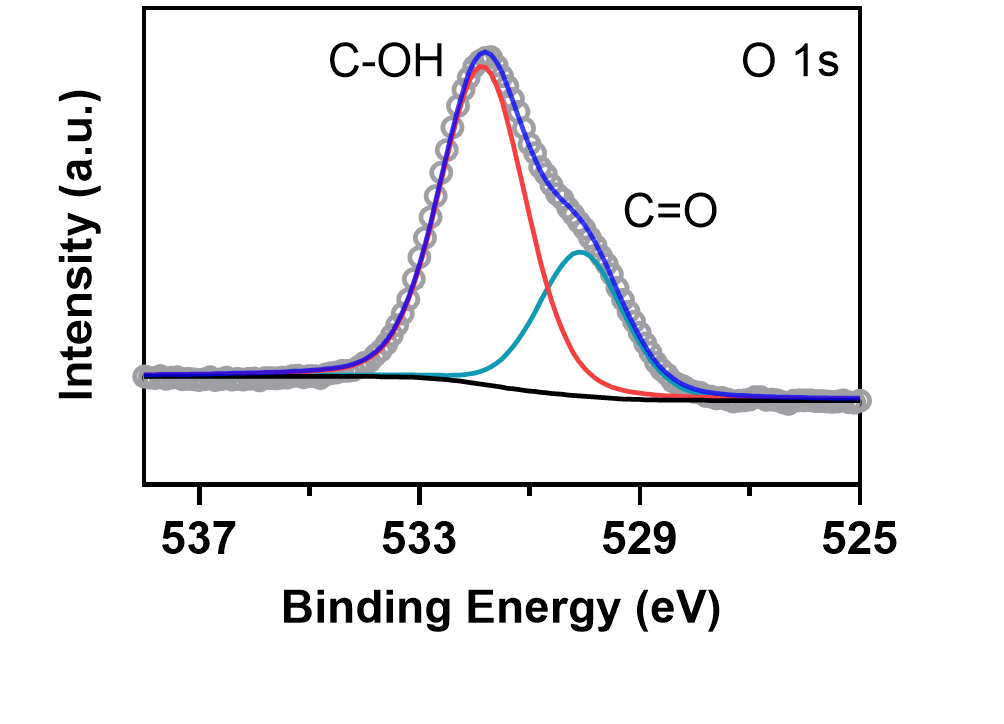


**Fig. S1**. O 1s XPS spectrum of PDA nanoparticles.


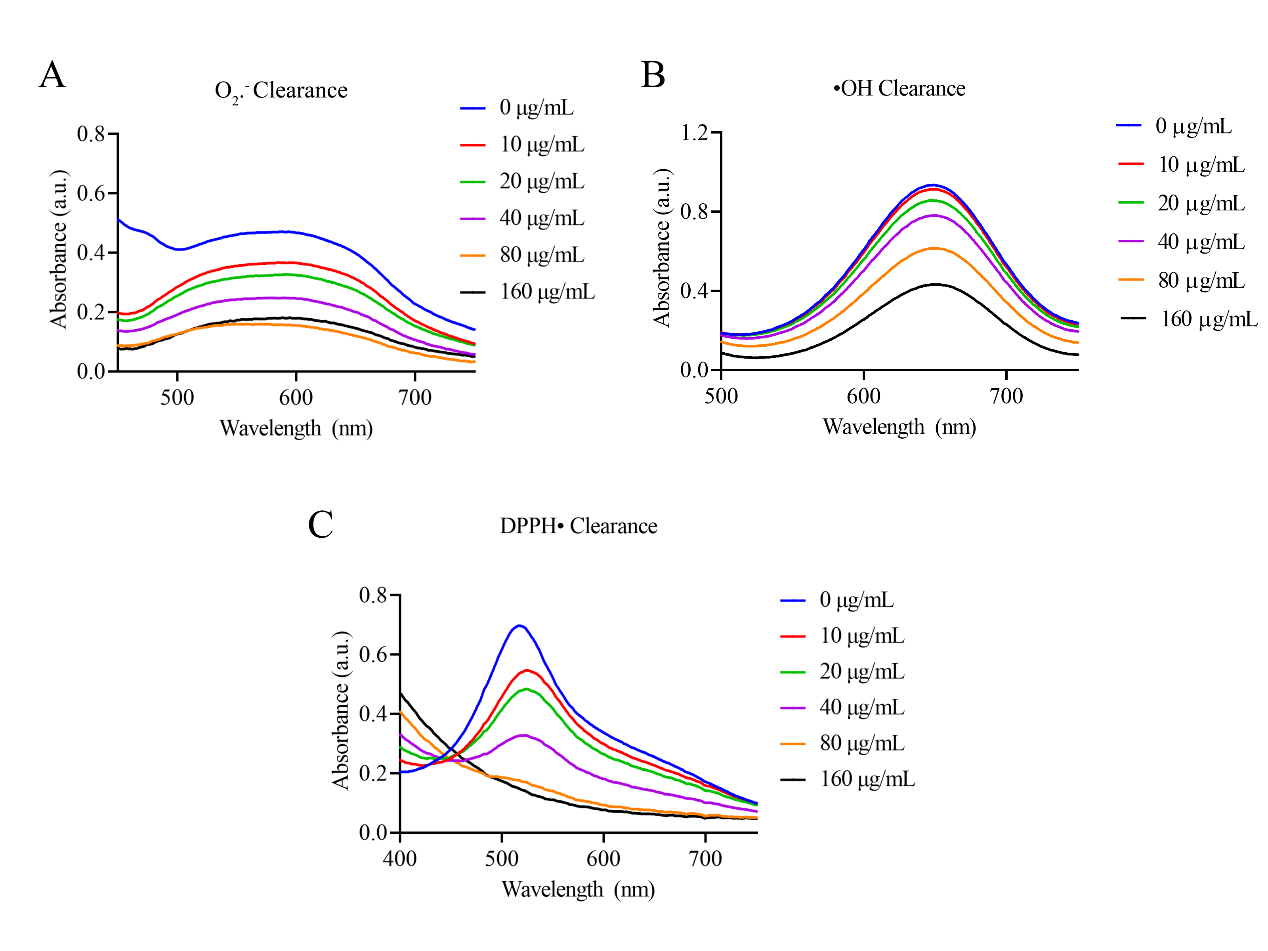


**Fig. S2.** Scavenging efficiencies of (A) superoxide anion (O_2_^•−^), (B) hydroxyl radicals (•OH), and (C) DPPH radical with different concentrations of PDA nanoparticles.


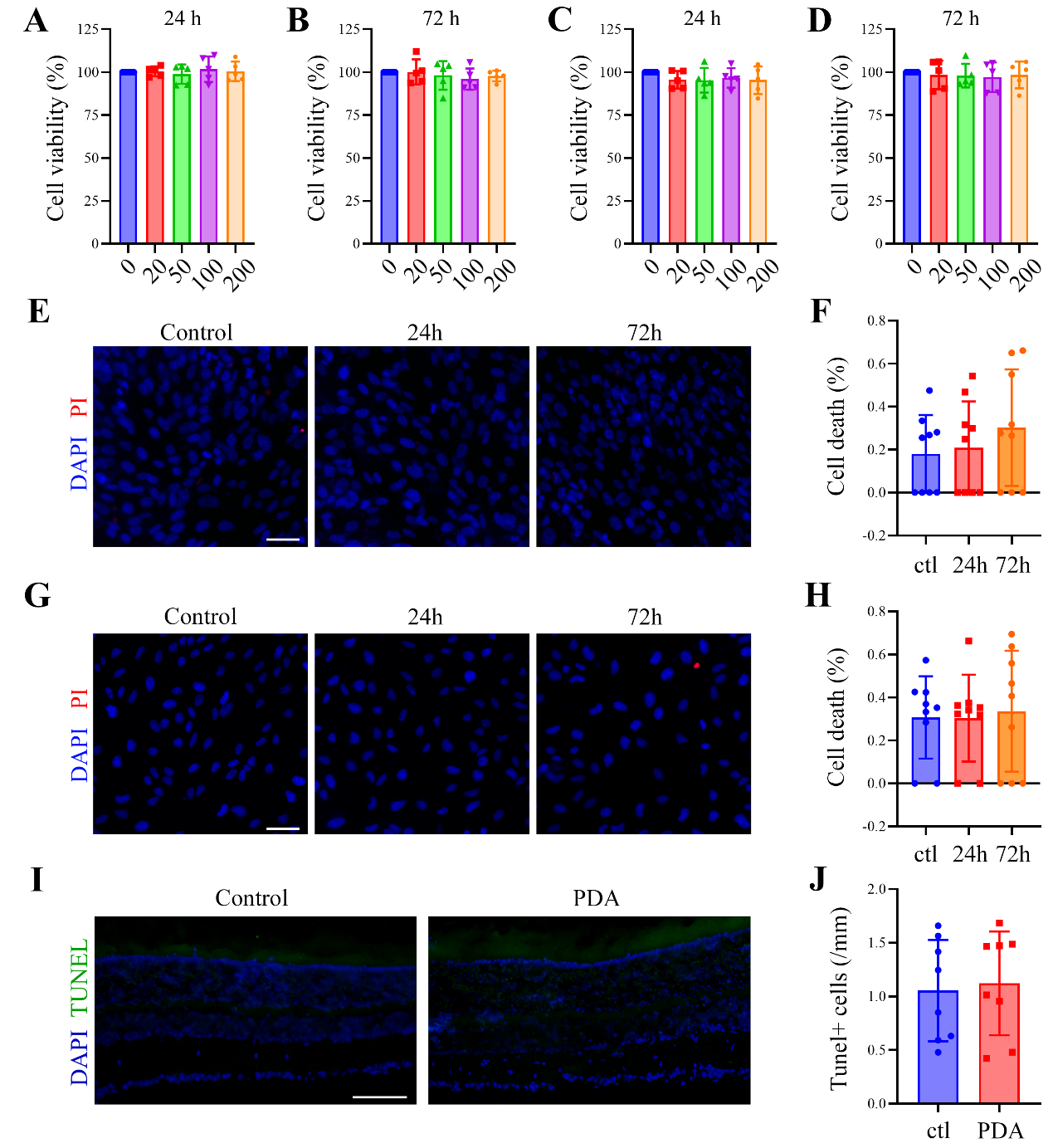


**Fig. S3.** Biocompatibility of PDA nanoparticles *in vitro* and *in vivo*. (A - D) The cell viability of 661W (A, B) and ARPE-19 (C, D) treated with different concentrations of PDA (0, 20, 50, 100 and 200 μg/mL) for 24 h and 72 h, which was determined by CCK-8 assay (n = 5). (E - H) Live/Dead cell staining of 661W (E, F) and ARPE-19 (G, H) cells treated with PDA (200 μg/mL) for 24 h or 72 h. Scale bar, 100 μm. n = 5. (I- J) PDA nanoparticles (4 μg) were intravitreously injected in the healthy mice, and the number of apoptotic cells in retinas is evaluated by TUNEL at day 7 post-injection. Scale bar, 50 μm. n = 8. Data are presented as the mean ± SD.


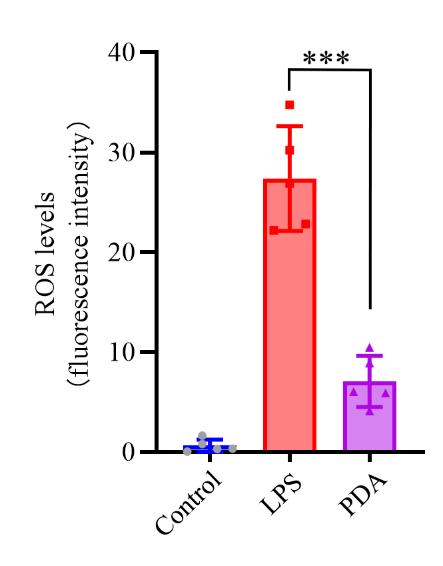


**Fig. S4.** Quantitative analysis (ANOVA) of ROS levels in Raw 264.7 treated with LPS (1 μg/mL) and PDA (200 μg/mL) for 12h. ****P* < 0.001. n = 5. Data are presented as the mean ± SD.


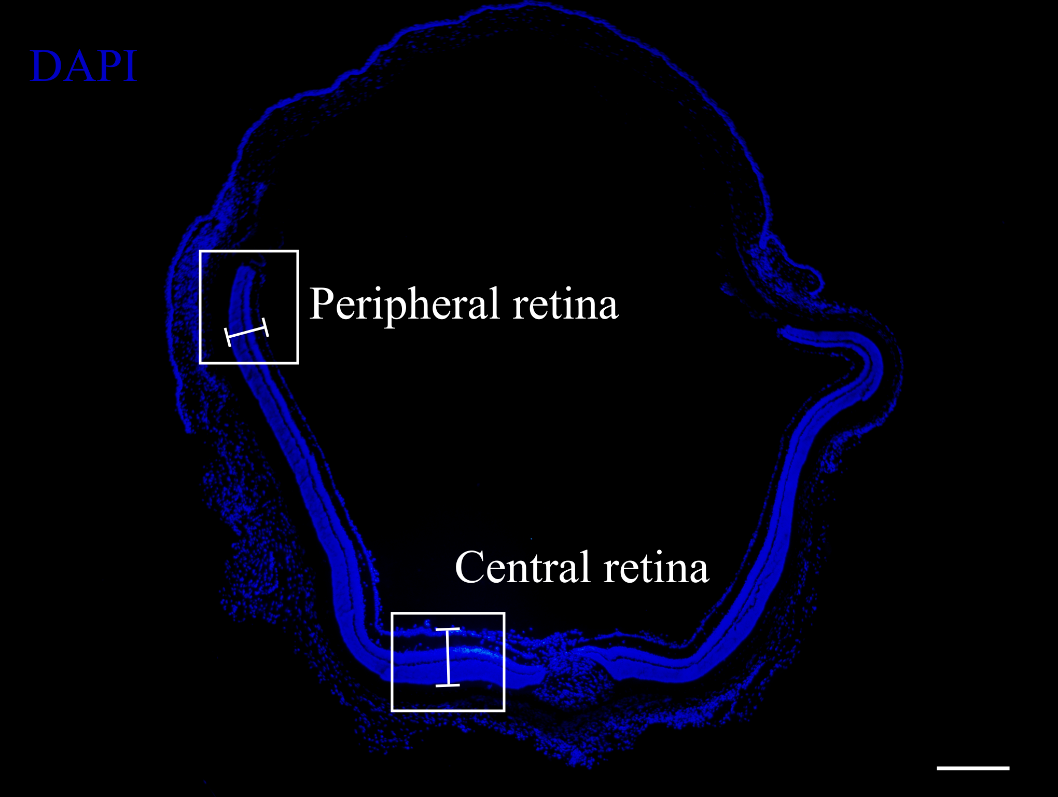


**Fig. S5.** DAPI staining of the retinal cross section, dashed lines indicate the region for analysis of central and peripheral retinal thickness. Scale bar, 200 μm.


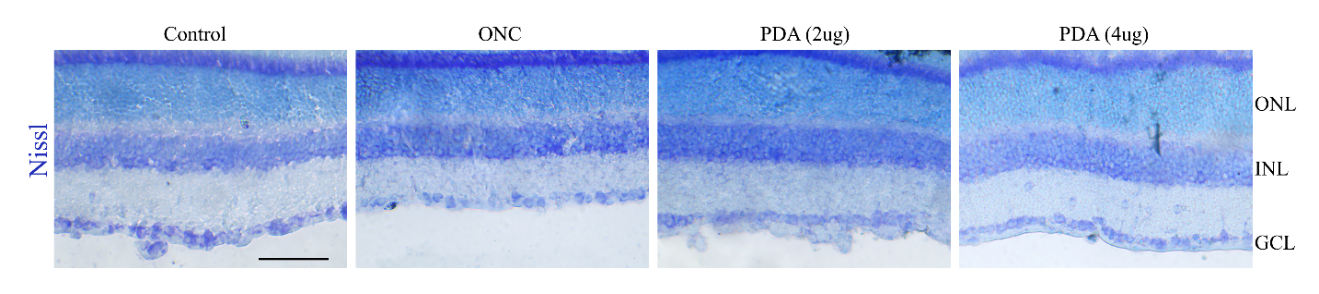


**Fig. S6.** Representative images of nissl stained retinal sections in mice treated with PDA (2 μg or 4 μg). Scale bar, 50 μm.


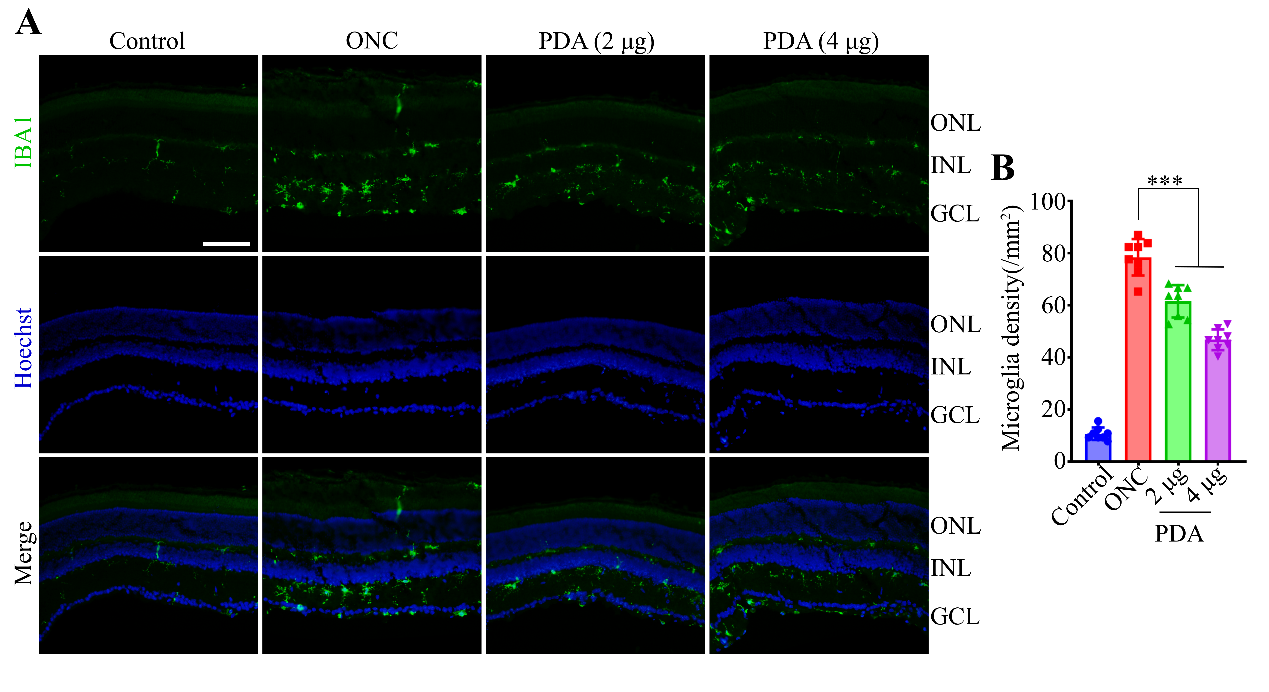


**Fig. S7.** Representative imageS and quantitative analysis (ANOVA) of microglia (IBA1-positive) densities in retinal sections. Scale bar, 50 μm. ****P* < 0.001. n = 8. Data are presented as the mean ± SD.


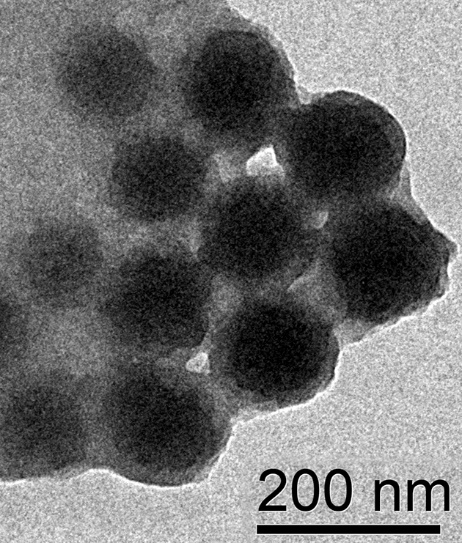


**Fig. S8.** TEM images of Br@PDA.
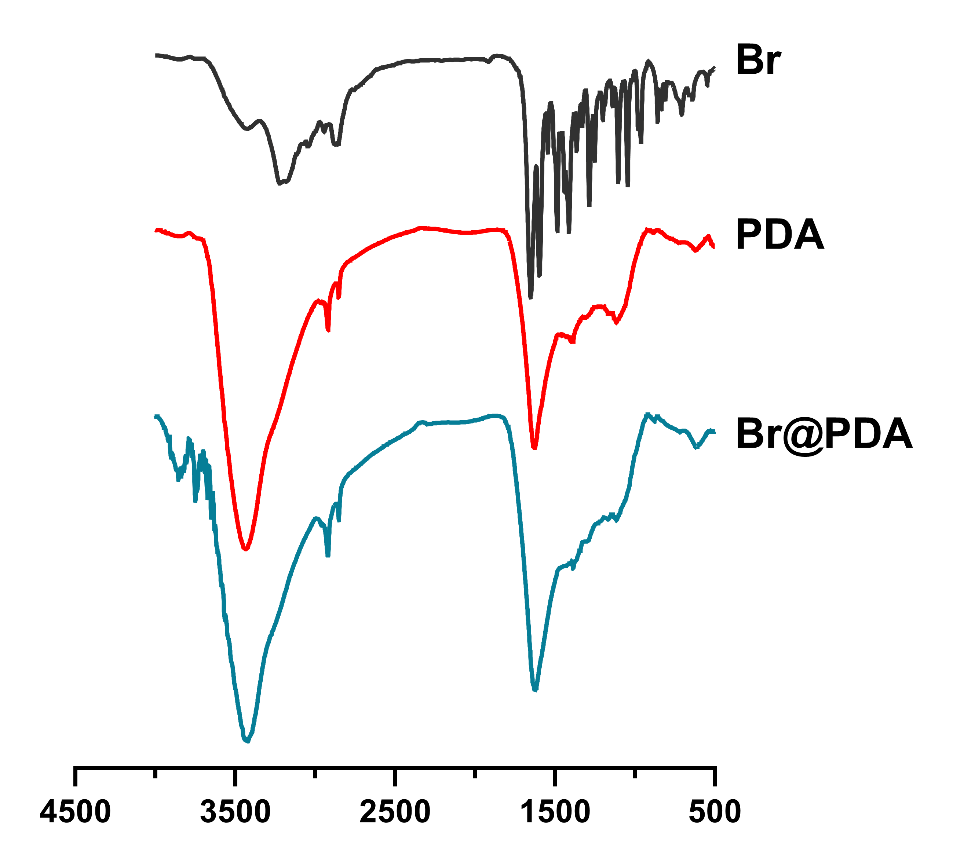


**Fig. S9.** FTIR spectra of Br, PDA and Br@PDA.


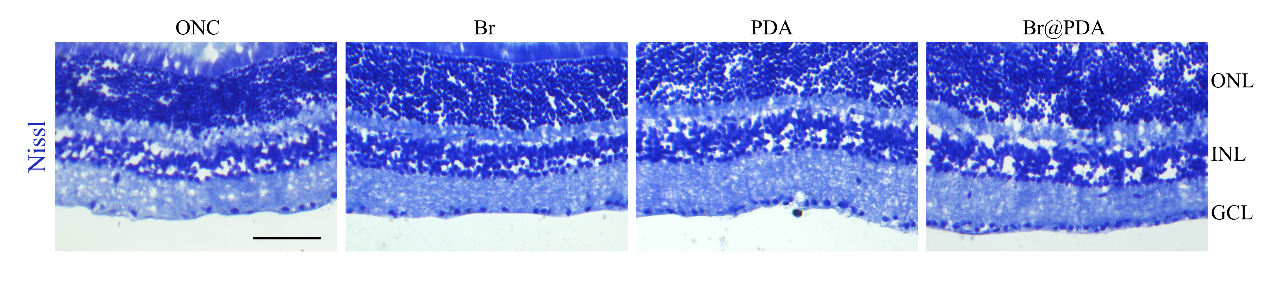


**Fig. S10.** Representative image of nissl stained retinal sections in mice treated with PDA, Br and Br@PDA. Scale bar, 50 μm.
